# A retrospective cohort study of open preperitoneal repair versus open suture repair for the treatment of emergency femoral hernia

**DOI:** 10.1038/s41598-020-60722-y

**Published:** 2020-02-28

**Authors:** Xiaochun Liu, Lujuan Ye, Guofu Zheng, Bo Ye, Weiqing Chen, Hailiang Xie, Yunqiang Liu, Yi Guo

**Affiliations:** Department of Vascular & Hernial Surgery, Ganzhou People’s Hospital (The Affiliated Ganzhou hospital of Nanchang University), No. 17, Red flag avenue, Ganzhou city, Jiangxi Province 341000 P.R. China

**Keywords:** Constipation, Outcomes research

## Abstract

To compare the outcomes of open preperitoneal repair (OPR) with the use of mesh and open suture repair (OSR) without mesh via inguinal approach for the treatment of emergency femoral hernia (FH). The primary outcome was the postoperative complication and the secondary outcomes were the recurrence rate of FH and the postoperative comfort level at the surgical site. 104 patients with emergency FHs were included, of whom 51 patients were treated with OPR, 53 patients were treated with OSR. Between the two groups, no significant difference was found in surgical site infection (SSI) (P = 0.801) or seroma (P = 0.843), while there was significant difference in the improvement of comfort at the surgical site (P = 0.013). The results of the 2-year follow-up demonstrated 1 and 8 cases of recurrence in the OPR and OSR group respectively, which was statistically significant (HR, 8.193 [95% CI, 1.024 to 65.547], P = 0.047). Compared with OSR, OPR with the use of mesh did not increase the risk of SSI and was safe to apply even under the condition of an emergency FH operation with intestine resection; OPR could reduce the recurrence rate of FH and improve the comfort at the surgical site.

## Introduction

The incidence of femoral hernia is not high, accounting for approximately 2–4% of all groin hernias^1,2^, but it is extremely easy for femoral hernias (FHs) to incarcerate and/or strangulate^3,4^, and patients need emergency surgery after incarceration and/or strangulation^5,6^. The surgical methods include tissue suture repair and tension-free repair with different kinds of meshes. Clinical guidelines do not strongly recommend tension-free repair with mesh because this approach may cause surgical site infection (SSI), especially in patients who undergo intestinal resection^7,8^. At present, with the improvement of mesh materials, there have been continuous reports of the safe use of mesh in the repair of incarcerated and/or strangulated groin hernias or even in the condition of intestinal resection without the occurrence of serious complications such as mesh-related infection^9–11^.

However, cohort studies comparing open mesh tension-free repair and open suture repair for emergency femoral hernias via the inguinal approach have rarely been reported. The purpose of this study was to evaluate the feasibility of open preperitoneal repair (OPR) for emergency FHs using UHS meshes by comparing the outcomes with those of open suture repair (OSR) for the treatment of emergency FHs.

## Patients and Methods

### Ethics statement

This retrospective cohort study was approved by the Medical Ethics Committee of Ganzhou People’s Hospital, and the methods were carried out in accordance with the approved guidelines.

### Patients

By referencing the medical records, consecutive patients with a primary diagnosis of FH treated between 2011 and 2017 were identified. Patients who underwent emergency FH repair via OPR with the use of UHS mesh or OSR without the use of mesh were selected and divided into two groups (the OPR group and OSR group) according to the repair method.

The inclusion criteria were as follows:

1. Age ≥ 18 years;
2. Emergency (incarcerated or strangulated) FH.

The exclusion criteria were as follows:

1. Patients did not undergo surgery after manual reduction;
2. The American Society of Anesthesiologists (ASA) score was too high for surgery;
3. Refused surgery;
4. Underwent laparoscopic repair.

Eligible patients’ personal and clinical details, including sex, age, body mass index (BMI), comorbidities, hernia position, duration of FH, duration of incarceration or strangulation, contents of the hernia sac, intestinal obstruction, and visual analogue scale (VAS) scores, were recorded at baseline. The duration of FH was defined as the time elapsed from when the lump appeared at the root of the thigh until admission, and the duration of incarceration or strangulation was defined as “the time elapsed from the start of incarceration or strangulation until surgery”.

## Methods

### Therapy options

According to the information provided in the informed consent form in the medical record, the therapy option was based on the preference expressed by either the patient or the treating clinician. The therapy options included OPR with the use of UHS mesh or OSR without the use of mesh.

Patients satisfying the inclusion criteria were fully informed of the OPR procedure with the use of UHS mesh or OSR without the use of mesh.

### Consent statement

Written informed consent was obtained pre-operation for each patient according to patients’ informed consent right of surgery.

### Surgical techniques

All patients underwent surgery performed by the same team. Patients were routinely given antibiotics during the perioperative period. Combined spinal-epidural analgesia or general anaesthesia was used for all procedures. After the skin and subcutaneous tissues in the inguinal area were incised, the spermatic cord or uterine round ligament was dissected. Next, the transverse fascia was opened, and gauze was used to protect the incision. Then, the hernia sac was dissected and opened. If the contents could not be reduced because of incarceration, part of the inguinal ligament and the iliopubic tract were incised, and the hernia sac was reduced into Hesselbach’s triangle over the inguinal ligament. The vitality of the hernia contents and the contamination of the hernia sac were then examined, and the fluid in the hernia sac was collected for bacterial culture.

Incarcerated hernia contents with normal vitality were reduced. If the strangulated intestines and/or omentum were necrotic, intestinal resection and anastomosis and omental resection were performed; then, the normal hernia contents were reduced.

Then, the hernia sac was closed. For the OSR group, the McWay repair was adopted. The conjoint tendon was sutured with the pectineal ligament.

For the OPR group, the preperitoneal space was further dissociated so that the lower layer of the UHS mesh covered the entire myopectineal orifice. Next, the dissected transverse fascia was sutured, and the upper layer of the mesh was subsequently fixed with absorbable sutures to the pubic tubercle and the inguinal ligament.

Finally, the severed inguinal ligament of the “OSR group” or the “OPR group” was repaired, and the external oblique aponeurosis was sutured. Subcutaneous drainage tubes were placed for postoperative continuous negative pressure drainage in patients with bowel resection and anastomosis.

### Follow-up protocol

All patients were followed on an outpatient basis and underwent physical examinations, duplex ultrasonography or telephone interviews. If clinically necessary, patients received more outpatient follow-up visits. The patients underwent re-examination at 1 month, 6 months and 12 months and then every year after the intervention. In this study, the 2-year follow-up results were recorded and analysed for both groups.

### Outcomes

The primary outcome was the postoperative complication such as seroma, superficial and deep SSI.

The secondary outcomes included the hernia recurrence rate (FH recurrence is defined as the reappearance of a reducible or non-reducible mass at the root of the thigh on the surgical side), and the comfort of the patient’s postoperative surgical site. Patient comfort was assessed with the VAS score.

### Statistical analysis

The continuous data of the two groups were described by means ± standard deviations (SDs), and their differences were compared with independent-samples *t*-tests. The categorical data were presented as percentages and compared with the χ2 test. The ranked data were analysed with a nonparametric test. Kaplan-Meier life tables and log-rank tests were applied to analyse the two groups in terms of the time to hernia recurrence rate, mortality and patients lost to follow-up.

All tests were two-sided with a significance level of 0.05 and were performed using SPSS software (ver. 22.0; IBM Corp., Armonk, NY, USA).

## Results

### General patient data

**Figure 1:**
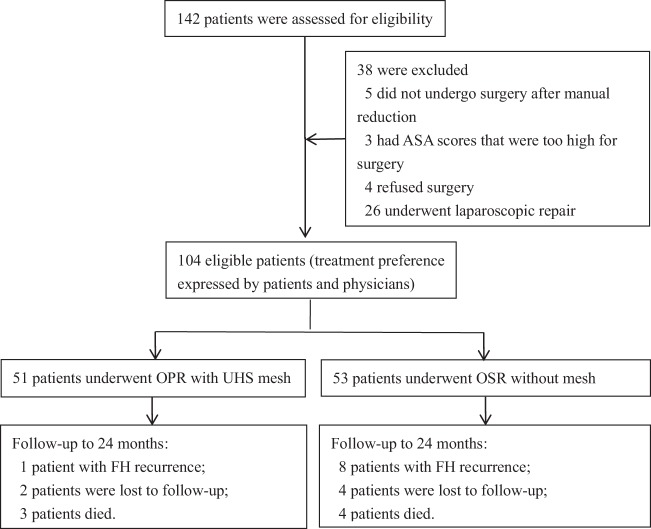
Assessment for Eligibility, Groups, and Outcomes. OPR, open preperitoneal repair; OSR, open suture repair; FH, femoral hernia; UHS, Ultrapro hernia system; ASA.

One hundred forty-two patients had emergency FHs and were treated in a single centre from 2011 to 2017. Thirty-eight patients were excluded, of whom 5 did not undergo surgery after manual reduction, 3 had an ASA score that was too high for surgery, 4 refused surgery, and 26 underwent laparoscopic repair. The number of eligible patients was 104 (Fig. 1). Based on the preference expressed by either the patient or the treating clinician, 51 patients were treated with OPR and UHS mesh, and the other 53 patients were treated with OSR. The baseline characteristics were similar in the two groups (Table 1). Factors considered to affect SSI and recurrence, such as age, BMI, comorbidity, duration of FH, duration of incarceration or strangulation, contents of the hernia sac, and intestinal obstruction, did not differ between the two groups.

**Table 1:** The baseline characteristics of patients in the study (More data in the suplementary file).

Variable	OPR group n = 51 (%)	OSR group n = 53 (%)	P value
Gender			
Male	19 (37.3)	14 (26.4)	0.235^§^
Female	32 (62.7)	39 (73.6)	
Year	72.4 ± 10.1	72.5 ± 13.0	0.952*
BMI	18.3 ± 2.5	18.9 ± 1.8	0.148*
Comorbidity			
Pulmonary infection	5 (9.8)	9 (17.0)	0.284^§^
Hypertension	7 (13.7)	6 (11.3)	0.711^§^
COPD	5 (9.8)	8 (15.1)	0.415^§^
Cardiac insufficiency	0 (0.0)	4 (7.5)	0.118^※^
Diabetes	3 (5.9)	0 (0.0)	0.114^※^
CHD	5 (9.8)	4 (7.5)	0.739^※^
Liver cirrhosis	2 (3.9)	3 (5.7)	1.0^※^
Malnutrition	6 (11.8)	8 (15.1)	0.619^§^
Location of the hernia			0.174^§^
Left	24 (47.1)	18 (34)	
Right	27 (52.9)	35 (66)	
Duration of FH (months)	47.9 ± 101.4	54.6 ± 81.8	0.709*
Duration of incarceration or strangulation (days)	5.0 ± 5.1	5.0 ± 4.1	0.996*
Contents of hernia sac			0.783^#^
Intestine	32 (62.7)	31 (58.5)	
Intestine and omentum	9 (17.6)	9 (17.0)	
Omentum	9 (17.6)	10 (18.9)	
Ileocecum	1 (2.0)	3 (5.7)	
Intestinal obstruction			0.459*
No	13 (25.5)	11 (20.8)	
Incomplete	6 (11.8)	5 (9.4)	
Complete	32 (62.7)	37 (69.8)	
VAS (Pre-operation)	86.7 ± 14.2	89.3 ± 11.5	0.312*

*Independent-sample *t*-test, ^§^Pearson Chi-Square, ^#^Likelihood Ratio, ^※^Fisher’s Exact Test, ^*^Mann-Whitney U test.

BMI, body mass index; COPD, chronic obstructive pulmonary disease; CHD, Coronary heart disease; FH, femoral hernia; VAS, Visual analog scale.

There was no significant difference between the two groups in terms of the overall treatments of the contents of the hernia sac (P = 0.163) (Table 2). Fifteen (29.4%) patients in the OPR group underwent intestinal resection, as did 24 (45.3%) patients in the OSR group (P = 0.095). The only difference was found in the treatment of reducing viable hernia content: 33 (64.7%) patients in the OPR group and 23 (43.4%) patients in the OSR group (P = 0.029). The results of the bacterial culture of liquid in the hernia sac were positive in 4 patients: one in the OPR group and the other 3 in the OSR group (P = 0.618). All the bacteria were *E. coli*.

**Table 2:** Procedure data, complications and follow-up outcomes (More data in the supplementary file).

Variable	OPR group n = 51 (%)	OSR group n = 53 (%)	P value
Management of hernia contents			0.163^#^
Intestinal resection	15 (29.4)^¤^	24 (45.3)^¤^	0.095^§^
Omental resection	5 (9.8)^¤^	8 (15.1)^¤^	0.415^§^
Intestinal and omental resection	2 (3.9)	2 (3.8)	1.0^※^
Hernia contents reduction	33 (64.7)	23 (43.4)	**0.029**^**§**^
Bacteria in hernia sac	1 (2.0)	3 (5.7)	0.618^※^
Complications			
Seroma	7 (13.7)	8 (15.1)	0.843^§^
Superficial SSI	5 (9.8)	6 (11.3)	0.801^§^
Deep SSI (including Mesh-related infection)	0 (0.0)	0 (0.0)	NA
Follow-up outcomes			0.051^#^
No recurrence	45 (88.2)	37 (69.8)	
Recurrence	1 (2.0)	8 (15.1)	
Death	3 (5.9)	4 (7.5)	
Lost to follow-up	2 (3.9)	4 (7.5)	

^¤^Including the patients of “Intestinal and omental resection”; *Independent-sample *t*-test; ^※^Fisher’s Exact Test; ^§^Pearson Chi-Square; ^#^Likelihood Ratio.

OPR, open preperitoneal repair; OSR, open suture repair; SSI, surgical site infection.

### Primary outcome

A certain number of complications occurred in both groups. Seven (13.7%) and 8 (15.1%) cases of seroma occurred in the OPR and OSR groups, respectively, with no significant difference (P = 0.843). In terms of infection, 5 (9.8%) cases of superficial SSI occurred in the OPR group, and 6 (11.3%) cases occurred in the OSR group (P = 0.801). However, no deep SSI (including mesh-related infection) occurred in either group (Table 2).

### Secondary outcomes

The results of the 2-year follow-up showed that 1 patient had FH recurrence in the OPR group, while 8 patients had FH recurrence in the OSR group. The overall recurrence rate was 8.7%. One patient in the OPR group had FH recurrence within half a month postoperatively. Six patients and 2 patients in the OSR group had FH recurrence within 1 year and 1–2 years postoperatively, respectively. During the 2 years of follow-up, 2 and 4 patients in the OPR and OSR groups were lost to follow-up, respectively. The mortality rate of emergency FH was relatively high. Three and 4 patients in the two groups died within 1 year postoperatively, among whom 3 patients died of pulmonary infection, 2 patients died of multiple organ failure, and 2 patients died of cardiac failure. Patients who died and those who were lost to follow-up were included in the censored cases for Kaplan-Meier curve analysis of the time to FH recurrence rate in the two groups.

**Figure 2:**
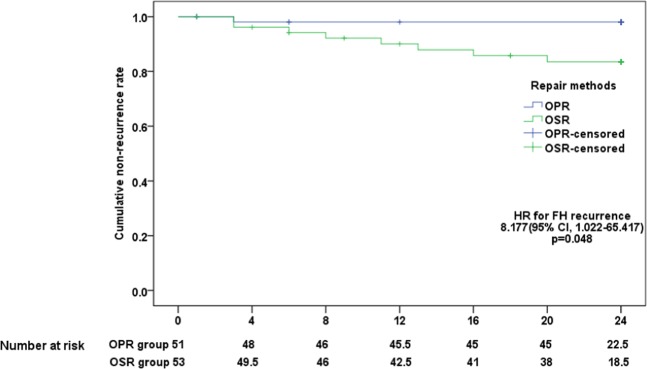
Kaplan–Meier Curves for Time to FH Non-recurrence in the Two Treatment Groups. FH, Femoral Hernia; OPR, open preperitoneal repair; OSR, open suture repair; HR, Hazard ratio; CI, Confidence interval.

The recurrence rate was significantly different between the two groups (hazard ratio [HR] for FH recurrence, 8.193 [95% CI, 1.024–65.547], P = 0.047) (Fig. 2). No significant differences were found between the two groups in terms of mortality (HR for mortality, 2.098 [95% CI, 0.384–11.462], P = 0.329) and lost to follow-up (HR for lost to follow-up, 1.298 [95% CI, 0.290–5.801], P = 0.733).

The VAS score was used to evaluate the comfort level of the surgical site in patients in the two groups preoperatively and 3 months postoperatively. The pre- and postoperative VAS scores of the patients in the two groups were significantly improved, but the improvement in the OPR group was significantly better than that in the OSR group (P = 0.013) (Table 3).

**Table 3:** Comparison of the VAS between the two groups (More data in the supplementary file).

	OPR group	OSR group	P value
Pre-operation	86.7 ± 14.2	89.3 ± 11.5	0.312*
3 months post-operation	4.6 ± 11.1	11.8 ± 17.1	0.013*
P value^&^ (Comparison of pre-operation and post-operation)	**0.000**	**0.000**	

*Independent-sample *t*-test; ^&^Paired samples test.

VAS, Visual analog scale.

## Discussion

Tension-free herniorrhaphy using mesh has become the gold standard for elective herniorrhaphy due to its low recurrence rate and improvement in comfort at the postoperative surgical site^7^. The treatment of incarceration and/or strangulated hernias, including FHs, begins with the removal of the incarceration and then herniorrhaphy to prevent recurrence. However, the use of mesh in the repair of incarcerated and/or strangulated hernias remains controversial due to the risk of SSI^11–13^. OPR and OSR for emergency FHs after intestinal resection and anastomosis have seldom been reported in the literature. This article is a retrospective cohort study of OPR versus OSR for the treatment of emergency FHs in our single centre.

The retrospective cohort study showed that compared with OSR, OPR with the use of UHS mesh did not increase the risk of SSI and was safe to apply even in the condition of emergency FH operations with intestine resection, and it improved the comfort at the surgical site. More importantly, the OPR method with the use of UHS mesh can reduce the recurrence rate of postoperative emergency FHs.

Hernia recurrence after emergency repair with a prosthetic mesh is a crucial issue for surgeons. Through this cohort study, we found that the FH recurrence rate was 1.96% (1 case) in the OPR group 2 years postoperatively, which was significantly lower than that in the OSR group (8 cases, 15.1%), and the overall recurrence rate was 8.7%.

The recurrence rate after FH surgery varies in different studies. In 2009, Swedish surgeons conducted a statistical analysis of patients with FHs in the Swedish national registry from 1992 to 2006 and found that the recurrence rate after emergency FH surgery was 7.4%^2^. Bessa *et al*.^10^. prospectively studied 234 patients who underwent emergency hernia repair using prosthetic meshes over a 10-year period, with an average follow-up of 62.5 ± 35.3 months, and found that only 2 patients experienced recurrence. In addition, the rate of recurrence of FH in two large national databases between 2005 and 2015 in the United States was between 5% and 6% in females and between 16% and 21% in males; the authors also found that there was a decrease in recurrence in females in one of the large national surveys^14^. However, the paper did not consider the method of repair. Andresen, Kristoffer *et al*.^15^ investigated the data on FH repairs registered in the Danish Hernia Database from January 1998 to February 2012. The authors analysed 3,970 patients who had FHs, of whom 511 patients underwent endoscopic repair, and 3459 patients underwent open repair; they found that endoscopic repair was associated with a lower recurrence rate than open repair, with a cumulative incidence of 0.62% vs 3.4%. However, they also found that the most frequently used method of open repair was plug repair, which was performed in 894 procedures, followed by the McVay repair (n = 186), which often resulted in increased recurrence.

All the different recurrence rates were significantly correlated with surgical status (emergency or elective), repair methods (laparoscopic repair, OPR or OSR), and the surgeon’s experience^2,9,14,16^. The focus of this study was open repair for emergency FHs, so the overall recurrence rate might be higher. However, the higher rate further indicates that OPR can significantly reduce the recurrence rate of emergency FHs.

In addition, the postoperative mortality rate of emergency FHs was relatively high. There were 3 cases and 4 cases of death in the OPR and OSR groups, respectively, within 1 year of postoperative follow-up; previous studies also supported this result^6,11^. There was no significant difference between the two groups for the comparison of death data, which did not affect the analysis results of the postoperative recurrence rate for FHs in this cohort study.

At present, the use of a mesh for the tension-free repair of an emergency hernia does not seem to be an absolute contraindication^11^. A mesh is safe to apply even in cases of intestinal resection and anastomosis^10,17–20^. Evidence has accumulated regarding the application of synthetic meshes, and clinicians recommend the use of synthetic meshes to repair FHs in patients, including in emergency situations^2,10,19^. One of the most important indicators of successful completion is the absence of mesh-related infection. In this cohort study, 15 (29.4%) and 24 (45.3%) patients underwent intestinal resection in the OPR and OSR groups, respectively, and no deep SSI occurred in either group. To prevent infection, during the perioperative period of emergency hernia surgery, antibiotics were empirically used; in addition, more importantly, gauze was always used to protect the wound and avoid contamination of the intestinal fluid during the operation. Furthermore, a drainage tube was placed under the skin without contacting the mesh. *E. coli* was indeed cultured from specimens of 1 and 3 patients in the OPR and OSR groups, respectively, via culturing of the hernia sac fluid. Some researchers have analysed the culture of bacteria in the incarcerated hernia sac and found that the most common bacteria in the hernia sac was *E. coli*^21^.

However, there were still 5 and 6 patients with superficial SSI in the OPR and OSR groups, respectively, and most of them recovered within 1 month after dressing changes. In addition, due to incarceration, a certain amount of postoperative seroma (7 cases vs 8 cases) occurred in both groups, but the seroma was well absorbed during the follow-up according to the ultrasound examination and did not develop into a deep SSI. Similar reports have been reported in the literature^10^. Seroma is a common complication after emergency hernia repair^9^.

UHS^22^ mesh was selected for OPR of FHs due to its bilayer polypropylene structure; another important reason for this approach was that the material of the UHS mesh is made of an absorbable polycarbon-25 coating and nonabsorbent polypropylene filament fibres that have antimicrobial properties^23^. These characteristics of the mesh may play a role in preventing deep SSIs including mesh-related infections.

The comfort at the surgical site is also an important indicator to evaluate postoperative effects. OPR for FHs can improve patient discomfort. From the comparison of the pre- and postoperative VAS scores of each group and the VAS scores 3 months postoperatively of the two groups, the postoperative comfort level at the surgical site was improved in both groups. However, the postoperative VAS scores of patients in the OPR group were significantly lower than those of patients in the OSR group, indicating that mesh repair could better improve postoperative comfort in patients with emergency FHs compared with suture repair. This should be related to the fact that OSR involves tension repair and OPR involves tension-free repair. The VAS scoring system is a good evaluation method for chronic pain after hernia surgery. In recent years, some studies have evaluated the postoperative effect of hernia using this evaluation system^24,25^.

Our study has several limitations. First, it involved a single centre and a relatively small number of patients. Second, this was a retrospective study performed using electronic medical records, which possibly introduced the potential for information bias. In addition, the therapy option was based on the preference expressed by either the patient or the treating clinician, which might have had some selection bias and effects on the results. Therefore, a multicentre, prospective randomized controlled study is necessary to evaluate the surgical effect of the UHS mesh for emergency tension-free repair of FHs.

In summary, the repair method of OPR with the use of UHS mesh did not increase the risk of SSI and was safe to apply, even in conditions where an emergency FH operation required intestine resection and anastomosis. OPR with the use of UHS mesh could reduce the recurrence rate of postoperative FH and improve the comfort at the surgical site in emergency FH treatment.

## Supplementary information

Supplementary information
